# What is the best way to organize vaccination services for the children of Quebec, Canada?

**DOI:** 10.1186/1472-6963-14-S2-P52

**Published:** 2014-07-07

**Authors:** Maryse Guav, Paule Clément, Carole Vanier, Sandie Briand, Cécile Michaud, Chantal Boulet, Joane Désilets, Fernand Guillemette, Eve Dubé, Nicole Boulianne, Jacques Lemaire, Monique Landry, Geneviève Baron

**Affiliations:** 1 University of Sherbrooke, Longueuil, Quebec, Canada; 2 National Institute of Public Health, Montreal, Quebec, Canada; 3 Charles LeMoyne Hospital Research Centre, Longueuil, Quebec, Canada; 4 Public Health Directorate, Agence de la sante et des services sociaux de la Montérégie, Longueuil, Quebec, Canada; 5 Public Health Directorate, Agence de la sante et des services sociaux de Lanaudière, Joliette, Quebec, Canada; 6 Public Health Directorate, Agence de la sante et des services sociaux de la Mauricie et du Centre-du-Québec, Trois-Rivières, Quebec, Canada; 7 Centre de recherche du CHU de Quebec, Quebec, Canada; 8 Quebec Ministry of Health, Montreal, Quebec, Canada; 9 Public Health Directorate, Agence de la santé et des services sociaux de I’Estrie, Sherbrooke, Quebec, Canada

## Background

Recently in Quebec, Canada, several contextual elements (e.g. physician disengagement, delayed appointments and late vaccines) justified a review of how child vaccination services are offered. A 5-year study begun in 2010-2011 aims to identify the optimal organizational model(s) for vaccination services for children aged 0-5 years. The first three year process and progress are reported.

## Materials and methods

This action research project adopts the Appreciative Inquiry methodology [1], using case studies [2]: 3 regions are currently studied (region 1: 16,000 births/year; region 2: 5,000 births/year; region 3:5,000 births/year). Building the model began with the development of a conceptual model drawn from a literature review. The participatory process relies on a steering committee for each case study, made up of players from local and regional levels and researchers. It meets monthly to discuss, reflect on and review vaccination service components. Various facilitation techniques foster the gradual production of an array of documents (e.g. accounts, schemas, tables) to fuel the discussion. Logbooks and field notes document the process. Qualitative analyses have been done.

## Results

To date, charts of service usage and schemas illustrating current vaccination service organization have been developed. The appointment-making processes, the functioning of vaccination clinics, the management of immunizing products and vaccination data have been described. Some courses of action have emerged and will be further explored: simplified appointment making, more systematic reminders and follow-ups, better structured vaccine transport and storage.

## Conclusion

Thanks to the approach used, emerging solutions will be more sustainable, acceptable and adapted to needs. Identification of the optimal model(s) for the organization of child vaccination services, adjusted to the various contexts is on progress.
